# Inhibition or knock out of Inducible nitric oxide synthase result in resistance to bleomycin-induced lung injury

**DOI:** 10.1186/1465-9921-6-58

**Published:** 2005-06-14

**Authors:** Tiziana Genovese, Salvatore Cuzzocrea, Rosanna Di Paola, Marco Failla, Emanuela Mazzon, Maria Angela Sortino, Giuseppina Frasca, Elisa Gili, Nunzio Crimi, Achille P Caputi, Carlo Vancheri

**Affiliations:** 1 Department of Clinical and Experimental Medicine and Pharmacology, Torre Biologica, Policlinico Universitario, 98123 Messina, Italy; 2 Department of Internal and Specialistic Medicine, Section of Respiratory Diseases, University of Catania, Catania, Italy; 3 Department of Experimental and Clinical Pharmacology, University of Catania, Catania, Italy

## Abstract

**Background:**

In the present study, by comparing the responses in wild-type mice (WT) and mice lacking (KO) the inducible (or type 2) nitric oxide synthase (iNOS), we investigated the role played by iNOS in the development of on the lung injury caused by bleomycin administration. When compared to bleomycin-treated iNOSWT mice, iNOSKO mice, which had received bleomycin, exhibited a reduced degree of the (i) lost of body weight, (ii) mortality rate, (iii) infiltration of the lung with polymorphonuclear neutrophils (MPO activity), (iv) edema formation, (v) histological evidence of lung injury, (vi) lung collagen deposition and (vii) lung Transforming Growth Factor beta1 (TGF-β1) expression.

**Methods:**

Mice subjected to intratracheal administration of bleomycin developed a significant lung injury. Immunohistochemical analysis for nitrotyrosine revealed a positive staining in lungs from bleomycin-treated iNOSWT mice.

**Results:**

The intensity and degree of nitrotyrosine staining was markedly reduced in tissue section from bleomycin-iNOSKO mice. Treatment of iNOSWT mice with of GW274150, a novel, potent and selective inhibitor of iNOS activity (5 mg/kg i.p.) also significantly attenuated all of the above indicators of lung damage and inflammation.

**Conclusion:**

Taken together, our results clearly demonstrate that iNOS plays an important role in the lung injury induced by bleomycin in the mice.

## Background

Pulmonary fibrosis is a progressive interstitial lung disease of unknown etiology. Pulmonary fibrosis is characterized by inflammatory cell infiltration, fibroblast proliferation, and excessive deposition of extracellular matrix proteins in the lung parenchyma [1,2]. The disease most commonly affects middle-age adults, although infants and children are also affected. Various studies have also indicated that the treatment with bleomycin during cancer chemotherapy in humans also induces interstitial fibrosis [3,4].

Nitric oxide (NO) is a pleiotropic mediator, which acts in a variety of physiological and pathophysiological processes [5-8]. NO is produced from the oxidation of L-arginine by the enzyme NO synthase [9,10] which occurs in three major isoforms; two are constitutive (endothelial and neuronal, indicated with cNOS), and one is inducible (macrophagic). The constitutively expressed enzyme (cNOS) are calcium-dependent, release NO under physiological condition in various cells, including endothelial cells and neurons, and NO released by these isoform are involved in the regulation of blood pressure in organ blood flow distribution, in the inhibition of the adhesion and activation of platelets and polymorphonuclear granulocytes and in neuronal transmission. The inducible isoform of NOS (iNOS) is calcium-independent and can be induced by proinflammatory agents, such as endotoxins (bacterial lipopolysaccharide, LPS), interleukin-1β, tumor necrosis factor-α (TNF-α) and interferon-γ (INF-γ), in endothelial and smooth-muscle cells, in macrophages and in other cell types [5-9]. Enhanced formation of NO following the induction of iNOS has been implicated in the pathogenesis of shock and inflammation [5].

Although the severity and duration of inflammation may dictate the timing and extent of NOS expression, it is now evident that the up-regulation of NOS can modulate inflammation [9-11]. Pharmacological inhibition of iNOS or genetic inactivation of NOS (iNOS knockout mice) attenuates the activation of the transcription factors nuclear factor kappa B (NF-κB) and signal transducer and activator of transcription-3 (STAT-3), and increases Granulocyte Colony-Stimulating Factor (G-CSF) messenger RNA levels in the tissue. Thus, induced nitric oxide, in addition to being a "final common mediator" of inflammation, is essential for the up-regulation of the inflammatory response. Furthermore, it has been recently suggested that some of the cytotoxic effects of NO are tightly related to the production of peroxynitrite, a high-energy oxidant deriving by the rapid reaction of NO with superoxide [12-14]. The resulting oxidative stress may cause cell death and tissue damage that characterize a number of human disease states like neurological disorders and stroke, inflammatory bowel disease, arthritis, toxic shock and acute reperfusion injuries [15-18]. Thus peroxynitrite, and not NO, has been proposed to be the ultimate cytotoxic species in many conditions acting through some mechanisms including the initiation of lipid peroxidation, the inactivation of a variety of enzymes (e.g. MnSOD) and the depletion of glutatione. Moreover, peroxynitrite is also able to induce DNA damage [19,20] resulting in inactivation of the nuclear enzyme PARS, in depletion of nicotinamide adenine dinucleotide (NAD+) and adenosine triphosphate (ATP) and lastly in cell death [21]. The realization of the cytotoxic potential of NO and peroxynitrite made it important to seek for pharmacological approaches, in order to neutralize NO and peroxynitrite-induced damage by inhibiting iNOS. The role of iNOS in pathologic condition have induced the development of selective iNOS inhibitors like GW274150 [(S)-2-Amino-(1-iminoethylamino)-5-thioheptanoic acid]. This molecule is a novel NOS-inhibitor (sulphur-substituted acetamine amono acid), which acts in competition with L-arginine and has a very high degree of selectivity for iNOS when compared to either eNOS (> 300-fold) or nNOS (> 100-fold) [22]. In addition GW274150 is a long acting (5 hours half life in rats) iNOS inhibitor and is also able to inhibit LPS-mediated increase in plasma NO_2_^- ^NO_3_^- ^levels 14 h after single intraperitoneal dose (ED50 3 mg kg^-1^) [23]. The inhibition of iNOS activity caused by GW274150 is NADPH-dependent and develops very slowly, but is rapidly reversible and recent studies reports the role of this iNOS selective inhibitors in reducing organ injury in hemorrhagic shock, in collagen induced arthritis and in renal ischemia/reperfusion [24-26]. In addition recently we have demonstrated that GW274150 treatment significantly reduced acute lung injury in an experimental model of carrageenan induced pleurisy [27]. Therefore the aim of this study was to investigate the role of iNOS in a model of lung injury induced by bleomycin administration using iNOSKO mice and iNOSWT mice. In addition, we have investigated the effects of the systemic administration of GW274150 in iNOSWT mice subjected to bleomycin-induced lung injury. In particular, we have investigated the effect of the genetic or pharmacological inhibition of iNOS on the bleomycin induced (i) loss of body weight, (ii) PMN lung infiltration [myeloperoxidase (MPO) activity], (iii) lung tissue edema [wet/dry ratio], (iv) lipid peroxidation, (v) the nitration of tyrosine residues (an indicator of the formation of peroxynitrite), (vi) lung damage (histology), (vii) lung collagen deposition and (viii) lung TGF-β1 expression.

## Materials and methods

### Animals

Male CD mice (25–35 g; Harlan Nossan; Italy) were housed in a controlled environment and provided with standard rodent chow and water. Animal care was in compliance with Italian regulations on protection of animals used for experimental and other scientific purpose (D.M. 116192) as well as with the EEC regulations (O.J. of E.C. L 358/1 12/18/1986).

### Experimental groups

Mice were randomly allocated into the following groups: (i) *iNOSWT + BLEO group*. Mice were subjected to bleomycin-induced lung injury (*N *= 30), (ii) *iNOSKO + BLEO group*. Mice were subjected to bleomycin-induced lung injury (*N *= 30), (iii) *iNOSWT +saline group*. Sham-operated group in which identical surgical procedures to the BLEO group was performed, except that the saline was administered instead of bleomycin, (iv) iNOSKO+*saline group*. Identical to *iNOSWT +saline group*, except for the use of *iNOSKO mice. GW274150 group*. Same as the *iNOSWT + BLEO group *but iNOSWT mice were administered with GW274150 (5 mg/kg) i.p. bolus 30 min after the administration of BLEO and every 24 h starting from day 1 (*N *= 30), (v) *Sham+ GW274150 group*. Identical to *iNOSWT +saline group*, except for the administration of GW274150 (5 mg/kg) i.p. bolus 30 min after the administration of BLEO and every 24 h starting from day 1 (*N *= 30). In another sets of studies, following bleomycin administration, the various groups of mice (*N *= 20 for each group) were observed for 15 days in order to determine survival differences. The dose of GW274150 used here has previously been reported by us to reduce the tissue injury caused by inflammation [26].

### Induction of lung injury by bleomycin

Mice received a single intratracheal instillation of saline (0.9%) or saline containing bleomycin sulphate (1 mg/kg body weight) in a volume of 50 μl and were killed after 15 days by pentobarbitone overdose.

### Measurement of fluid content in lung

The wet lung weight was measured after careful excision of extraneous tissues. The lung was exposed for 48 h at 180°C and the dry weight was measured. Water content was calculated by subtracting dry weight from wet weight.

### Histological examination

Lung biopsies were taken 15 days after injection of bleomycin. Lung biopsies were fixed for 1 week in 10% (w/v) PBS-buffered formaldehyde solution at room temperature, dehydrated using graded ethanol and embedded in Paraplast (Sherwood Medical, Mahwah, NJ, USA). After embedding in paraffin, the sections were prepared and stained by H&E or by trichrome stain. All sections were studied using light microscopy (Dialux 22 Leitz). The severity of fibrosis was semi quantitatively assessed according to the method proposed by Ashcroft and co-workers [28]. Briefly, the grade of lung fibrosis was scored on a scale from 0 to 8 by examining section randomly chosen fields per sample at a magnification of ×100. Criteria for grading lung fibrosis were as follows: grade 0, normal lung; grade 1, minimal fibrous thickening of alveolar or bronchiolar walls; grade 3, moderate thickening of walls without obvious damage to lung architecture; grade 5, increased fibrosis with definite damage to lung structure and formation of fibrous bands or small fibrous masses; grade 7, severe distortion of structure and large fibrous areas; grade 8, total fibrous obliteration of fields.

### Collagen Protein Measurement

Total lung collagen content was measured by means of Sircol Soluble Collagen Assay (Biocolor, Newtownabbey, Northern Ireland), an assay based on a modification of the sirius red method, as recommended by the manufacturer. Briefly, after the sacrifice, mice lungs were explanted and homogenized. Samples were then incubated at 4°C for 2 h and centrifuged at 15,000 × g. Supernatants (20 μl) were diluted 5 times in lysis buffer, added to 1 mL of Sircol Dye Reagent and then mixed for 30 minutes at room temperature in a mechanical shaker. The collagen-dye complex was precipitated by centrifugation at 10000 × g for 10 min. The unbound dye solution was then carefully removed. The precipitated complex was resuspended in 1 mL of alkali reagent. The obtained solution was finally placed in a 96 wells flat bottomed plate and evaluated in a plate reader (absorbance = 540 nm). Obtained values were then compared to the standard curve as recommended to obtain absolute collagen content. Shown data represent the mean collagen content, expressed as μg/μl of lung homogenates (± SE), of at least 4 independent experiments.

### Immunohistochemical localization of nitrotyrosine

Tyrosine nitration, an index of the nitrosylation of proteins by peroxynitrite and/or ROS, was determined by immunohistochemistry as previously described [29]. At the end of the experiment, the tissues were fixed in 10% (w/v) PBS-buffered formaldehyde and 8 μm sections were prepared from paraffin embedded tissues. After deparaffinization, endogenous peroxidase was quenched with 0.3% (v/v) hydrogen peroxide in 60% (v/v) methanol for 30 min. The sections were permeablized with 0.1% (w/v) Triton X-100 in PBS for 20 min. Non-specific adsorption was minimized by incubating the section in 2% (v/v) normal goat serum in PBS for 20 min. Endogenous biotin or avidin binding sites were blocked by sequential incubation for 15 min with biotin and avidin (DBA, Milan, Italy), respectively. Sections were incubated overnight with anti-nitrotyrosine polyclonal antibody (1:500 in PBS, v/v). Sections were washed with PBS, and incubated with secondary antibody. Specific labeling was detected with a biotin-conjugated goat anti-rabbit IgG and avidin-biotin peroxidase complex (DBA, Milan, Italy). In order to confirm that the immunoreactions for the nitrotyrosine were specific some sections were also incubated with the primary antibody (anti-nitrotyrosine) in the presence of excess nitrotyrosine (10 mM) to verify the binding specificity. In this situation no positive staining was found in the sections indicating that the immunoreactions were positive in all the experiments carried out.

### Myeloperoxidase activity

Myeloperoxidase (MPO) activity, an indicator of polymorphonuclear leukocyte (PMN) accumulation, was determined as previously described [30]. At the specified time following injection of bleomycin, lung tissues were obtained and weighed, each piece homogenized in a solution containing 0.5% (w/v) hexadecyltrimethyl-ammonium bromide dissolved in 10 mM potassium phosphate buffer (pH 7) and centrifuged for 30 min at 20,000 × g at 4°C. An aliquot of the supernatant was then allowed to react with a solution of tetramethylbenzidine (1.6 mM) and 0.1 mM hydrogen peroxide. The rate of change in absorbance was measured spectrophotometrically at 650 nm. MPO activity was defined as the quantity of enzyme degrading 1 μmol of peroxide/min at 37°C and was expressed in milliunits per g of wet tissue.

### Thiobarbituric acid-reactant substances measurement

Thiobarbituric acid-reactant substances measurement, which is considered a good indicator of lipid peroxidation, was determined, as previously described [27], in the lung tissues. At the specified time following injection of bleomycin lung tissues were homogenized in 1.15% KCl solution. An aliquot (100 μl) of the homogenate was added to a reaction mixture containing 200 μl of 8.1% SDS, 1500 μl of 20% acetic acid (pH 3.5), 1500 μl of 0.8% thiobarbituric acid and 700 μl distilled water. Samples were then boiled for 1 h at 95°C and centrifuged at 3,000 × g for 10 min. The optical density at 650 nm (OD_650_) was measured using ELISA microplate reader (SLT- Labinstruments Salzburg, Austria). Thiobarbituric acid-reactant substances were calculated by comparison with OD_650 _of standard solutions of 1,1,3,3-tetramethoxypropan 99% malondialdehyde bis (dymethyl acetal) 99% (Sigma, Milan). The absorbance of the supernatant was measured by spectrophotometry at 650 nm.

### Bronchoalveolar Lavage (BAL)

Seven days after bleomycin or saline solution instillation, mice were euthanized and the trachea was immediately cannulated with an I.V. polyethylene catheter (Neo Delta Ven 2, delta Med, Viadana, Italy) equipped with a 24-gauge needle on a 1 mL syringe. Lungs were lavaged once with 0.5 ml D-PBS (GIBCO, Paisley, U.K.). In >95% of the mice, the recovery volume was over 0.4 ml. The BAL fluid was spun at 800 rpm, the supernatant was removed and the pelleted cells were collected. Total BAL cells were enumerated by counting on a hemocytometer in the presence of trypan blue. Cytospins were prepared from resuspended BAL cells.

Cytospins of BAL cells were made by centrifuging 50,000 cells onto microscope slides using a Shandon Cytospin 3 (Shandon, Astmoore, U.K.). Slides were allowed to air dry and were then stained with Diff-Quick Stain Set (Diff-Quick; Baxter Scientific, Miami, FL). A total of 400 cells were counted from randomly chosen high power microscope fields for each sample. The differential percentage was multiplied by the total leukocyte number per mL to derive the absolute number of monocyte/macrophages, neutrophils, lymphocytes and eosinophils.

### TGF-β1 western blot analysis

Immediately after sacrifice, lungs were removed, thoroughly washed, frozen and stored at -80°C until protein extraction. Thawed tissues were washed in PBS and homogenized with an Ultra-Turrax T25 tissue grinder in 400 μl of 10 mM cold Tris homogenization buffer containing 5 mM EDTA, 1% Tryton-X100, 1 mM phenylmethylsulfonylfluoride, 25 μg/ml leupeptin and 0.5% aprotinin (all from Sigma-Aldrich). After homogenization, samples were incubated at 4°C for 2 h, centrifuged for 10 min at 15,000 × g and the supernatant was processed for protein concentration according to the method of Bradford [31]. Samples were diluted in sample buffer and boiled for 5 min. Electrophoresis was performed in 15% sodium dodecyl sulfate (SDS)-polyacrylamide gel electrophoresis (40 mA/h) using 60–80 μg of cell proteins per lane. After separation, proteins were transferred onto a nitrocellulose membrane (Hybond ECL, Amersham Biosciences Europe GmbH, Milan, Italy) for 2 h at room temperature using a transblot semidry transfer cell. After blocking, the membranes were incubated with a monoclonal mouse anti-TGF-β1 (0.8 μg/ml; Chemicon, Temecula, CA) overnight at 4 °C. Membranes were then thoroughly washed and incubated with HRP-conjugated secondary antibody. Specific bands were visualized using the SuperSignal chemiluminescent detection system (Pierce Biotechnology Inc., Rockford, IL). The same membranes were washed with a stripping solution containing 0.2 M glycine, 0.1% SDS, 1% Tween-20 and re-blotted with mouse anti-β-actin (1:250; Sigma-Aldrich) overnight at 4°C and processed for signal detection as described above.

TGF-β1 expression was normalized for β-actin and data are expressed as the percent increase of TGF-β1 expression in bleomycin-treated mice vs. control animals.

### Materials

Unless otherwise stated, all compounds were obtained from Sigma-Aldrich Company Ltd. (Poole, Dorset, U.K.). All other chemicals were of the highest commercial grade available. All stock solutions were prepared in non-pyrogenic saline (0.9% NaCl; Baxter, Italy, UK).

### Statistical evaluation

All values in the figures and text are expressed as mean ± standard error of the mean (SEM) of N observations. For the in vivo studies N represents the number of animals studied. In the experiments involving histology or immunohistochemistry, the figures shown are representative of at least three experiments performed on different experimental days. The results were analyzed by one-way ANOVA followed by a Bonferroni *post-hoc *test for multiple comparisons. A P-value of less than 0.05 was considered significant. Statistical analysis for survival data was calculated by Fisher's exact probability test. For such analyses, *p *< 0.05 was considered significant. Mann-Withney U test was used to compare the percent increase of TGF-β1 in iNOS^-/- ^and GW274150-treated animals versus iNOS wild type mice; a *p *< 0.05 was considered significant.

## Results

### The development of bleomycin-induced lung injury is attenuated in iNOSKO mice

**Figure 1:**
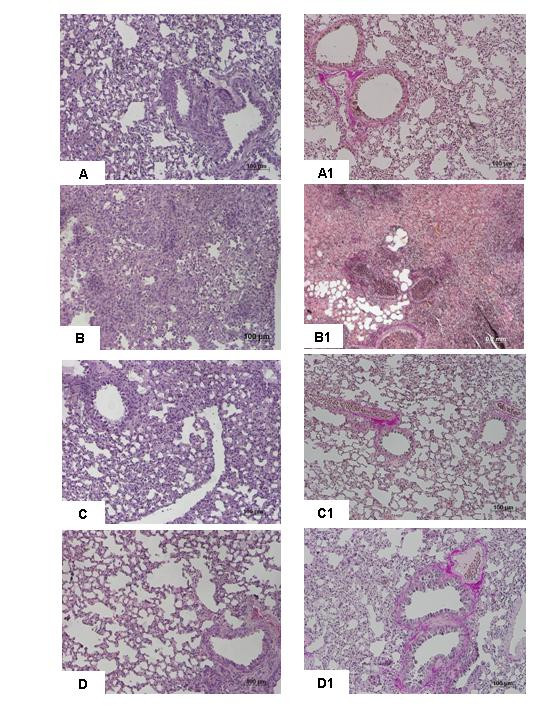
**Effect of iNOS inhibition on lung injury**. H&E stain: × 250. **A: **Saline control, normal lung architecture. **B: **Bleomycin alone in iNOSWT mice, extensive inflammation with inflammatory cells infiltration and fibrosis. **C: **Bleomycin in iNOSKO mice patchy areas of inflammation with minimal fibrosis. **D: **Bleomycin in iNOSWT mice plus GW274150 patchy areas of inflammation with minimal fibrosis. Comparable sections of mouse lung stained with trichrome: **A1: **saline control: normal lung architecture; **B1**; Bleomycin alone in iNOSWT mice, extensive areas of collagen; **C1: **Bleomycin in iNOSKO mice minimal collagen; **D1: **Bleomycin in iNOSWT mice plus GW274150 minimal collagen. Figure is representative of at least 3 experiments performed on different experimental days.

**Figure 2:**
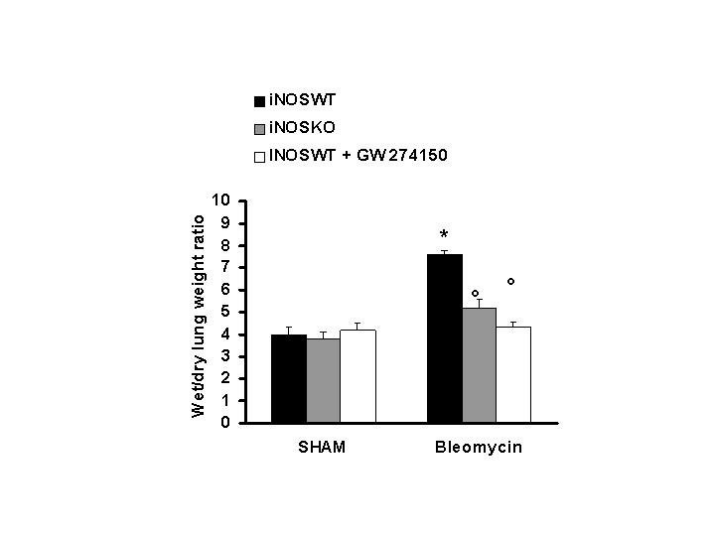
**Effect of genetic or pharmacological inhibition of iNOS on edema in the lung**. The injection of bleomycin in iNOSWT mice elicited an inflammatory response characterized by the accumulation of water in lung as an indicator of edema. The genetic or pharmacological inhibition of iNOS significantly reduced the edema formation. Data are means ± s.e. means from 10 mice for each group. *p < 0.01 versus sham. °p < 0.01 represents significant reduction of the various parameters in the group in which iNOS was inhibited or absent.

**Figure 3:**
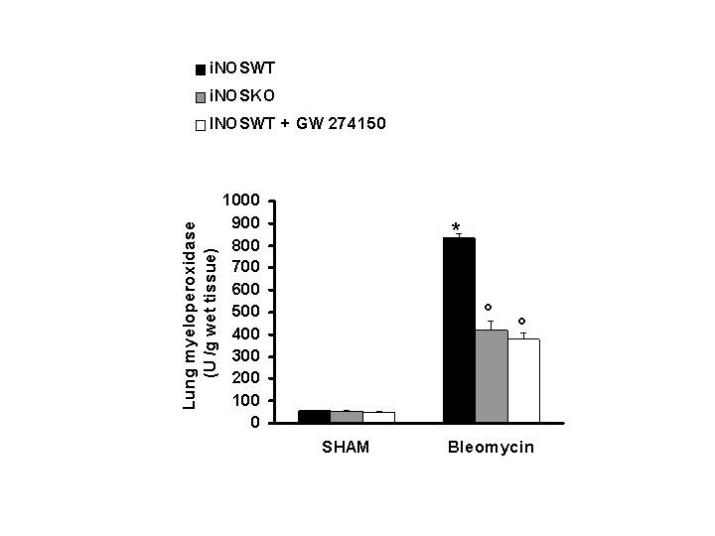
**Effect of genetic or pharmacological inhibition of iNOS on myeloperoxidase activity in the lung**. Myeloperoxidase (MPO) activity in the lungs of bleomycin-treated iNOSWT mice were significantly increased in comparison to sham-operated mice. The genetic or pharmacological inhibition of iNOS significantly reduced the bleomycin-induced increase in MPO activity. Data are means ± s.e. means from 10 mice for each group. *p < 0.01 versus sham. °p < 0.01 represents significant reduction of the various parameters in the group in which iNOS was inhibited or absent.

Histological examination of lung sections revealed significant tissue damage (Fig 1B,B1 Table 1). Thus, when compared to lung sections taken from saline-treated animals (Fig. 1A,A1 Table 1), histological examination of lung sections of iNOSWT mice treated with bleomycin characterized by extensive inflammatory infiltration by neutrophils, lymphocyte and plasma cells extending through the lung epithelial (Fig. 1B,B1 Table 1), fibrosis (Fig. 1B1 Table 1) and granulomas in perivascular region (Fig. 1B1). The absence or inhibition of iNOS in mice (animals with the iNOSKO phenotype or iNOSWT mice treated with GW274150) significantly prevented lung inflammation induced by bleomycin administration (Fig. 1C,C1, D,D1 respectively). Furthermore, the injection of bleomycin in iNOSWT mice elicited an inflammatory response characterized by the accumulation of water in lung as an indicator of fluid content, (Fig. 2) and neutrophils infiltration in the lung tissues (Fig. 3). The absence or inhibition of iNOS in mice (animals with the iNOSKO phenotype or iNOSWT mice treated with GW274150) significantly reduced the fluid content and the neuthrophil infiltration (Figs. 2, 3).

**Table 1:** Histological Scoring of lung fibrosis

	**SHAM + vehicle**	**Bleomycin + iNOSWT**	**Bleomycin + iNOSKO**	**Bleomycin + iNOSWT + GW274150**
	
**Lung fibrosis score**	ND	5.1 ± 0.11*	1.5 ± 0.08°	1.4 ± 0.10°

The above parameters were evaluated at 15 days after bleomycin administration. *p < 0.01 versus sham. °p < 0.01 represents significant reduction of the various parameters in the group in which iNOS was inhibited or absent.

### iNOSKO and GW274150 treated mice show a reduced collagen production in response to bleomycin

**Figure 4:**
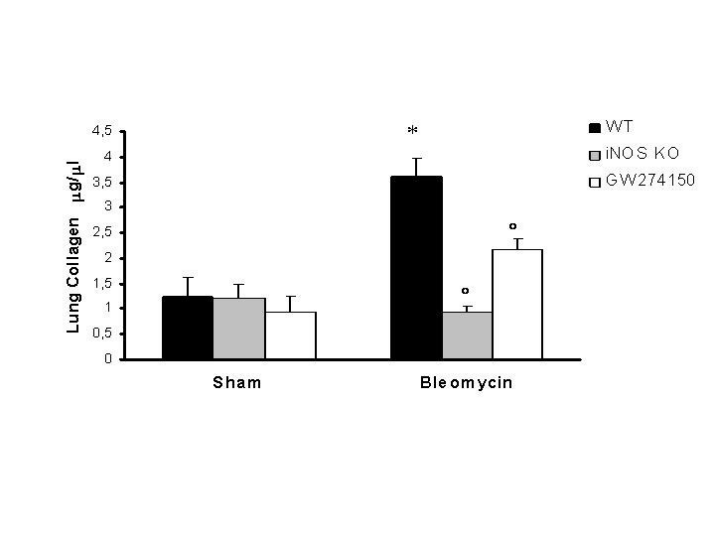
**Data represent the mean collagen content, expressed as μg/μl of lung homogenates (+/- SE), of at least 4 independent experiments**. *p < 0.01 versus sham. °p < 0.01 represents significant reduction of the various parameters in the group in which iNOS was inhibited or absent.

iNOSWT mice exposed to bleomycin showed a significant increase of lung collagen content after 7 days if compared to sham mice: from 1.23 ± 0.39 μg/μl to 3.62 ± 0.33 μg/μl, p < 0.001. iNOSKO and GW274150 treated iNOSWT mice that underwent bleomycin tracheal instillation did not show such an increase of lung collagen content (Fig 4). These animals, when exposed to bleomycin, showed a reduced collagen lung deposition if compared to iNOSWT mice: 0.94 ± 0.12 and 2.18 ± 0.17 μg/μl vs. 3.62 ± 0.33 μg/μl, (p < 0.001 and p < 0.01 respectively, Fig. 4)

### Nitrotyrosine formation and lipid peroxidation

**Figure 5:**
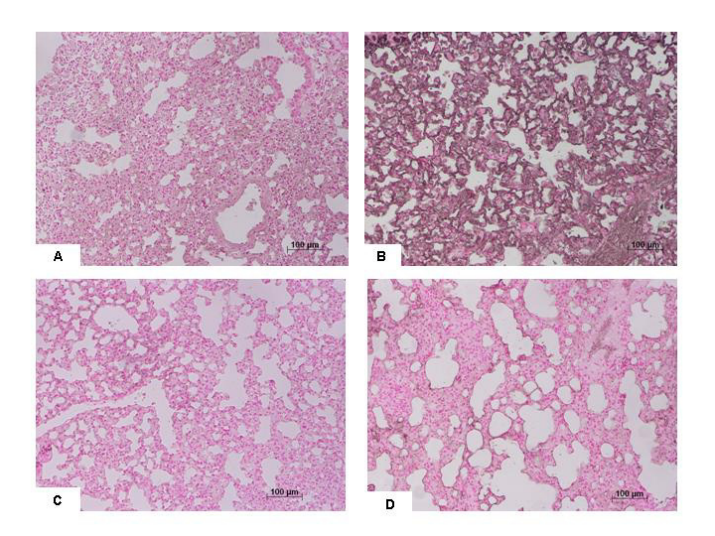
**Immunohistochemical localization of nitrotyrosine in the lung**. No positive staining was observed in the lung section for sham-treated mice (**A**). After bleomycin injection in iNOSWT mice, positive staining for nitrotyrosine (**B**) was localized mainly in nuclei of inflammatory cells. There was a marked reduction in the immunostaining in the lungs of bleomycin-treated iNOSKO mice (**C**) and in the lungs of bleomycin-treated iNOSWT mice which received GW274150 (**D**). Original magnification: 150×. This figure is representative of at least 3 experiments performed on different experimental days.

**Figure 6:**
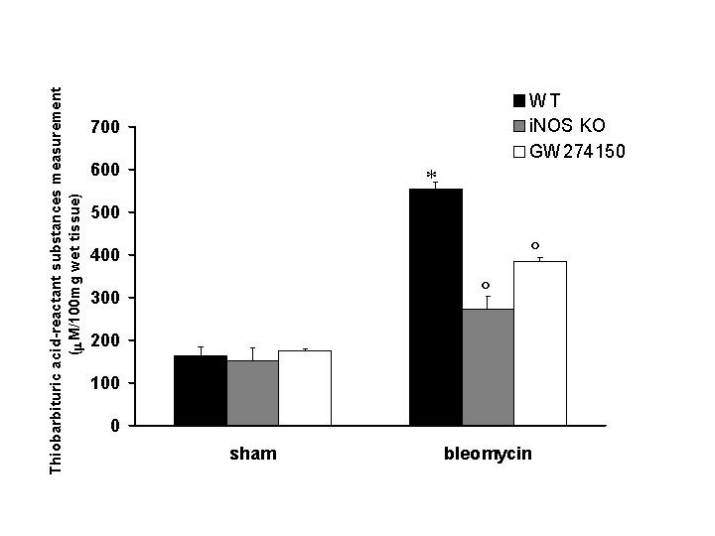
**Effect of genetic or pharmacological inhibition of iNOS on Thiobarbituric acid-reactant substances, a good indicator of lipid peroxidation, in the lung**. Thiobarbituric acid-reactant substances levels in the lungs of bleomycin-treated iNOSWT mice were significantly increased in comparison to sham-operated mice. The genetic or pharmacological inhibition of iNOS significantly reduced the bleomycin-induced increase in Thiobarbituric acid-reactant substances levels. Data are means ± s.e. means from 10 mice for each group. *p < 0.01 versus sham. °p < 0.01 represents significant reduction of the various parameters in the group in which iNOS was inhibited or absent.

Immunohistochemical analysis of lung sections obtained from bleomycin-treated iNOSWT mice revealed a positive staining for iNOS manly localized in plasma cell and lymphocytes (Fig. 5B). In contrast, no staining for iNOS was found in the lungs of bleomycin-treated iNOSKO mice (Fig. 5C) and in the lung from bleomycin-injected iNOSWT mice treated with GW274150 (Fig. 5D). Staining was absent in lung tissue obtained from the sham group (Fig. 5A). All iNOSWT mice, who were treated with bleomycin, exhibited a substantial increase in the lung thiobarbituric acid-reactant substances levels (index of lipid peroxidation) (Fig. 6). The absence or inhibition of iNOS in mice (animals with the iNOSKO phenotype or iNOSWT mice treated with GW274150) significantly attenuate the increase in thiobarbituric acid-reactant substances lung levels caused by bleomycin in the lung (Fig. 6). There was no increase in lung thiobarbituric acid-reactant substances level in sham-operated animals (Fig 6).

### Effect of INOS inhibition on the on changes of body weight and survival rate

**Figure 7:**
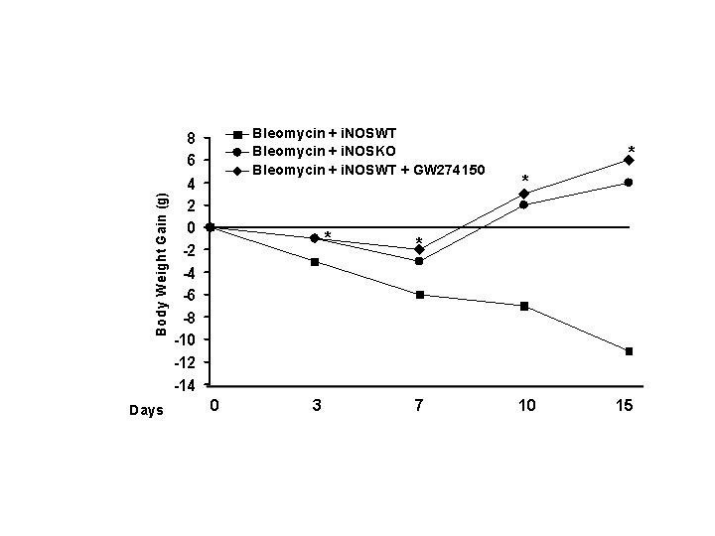
**Effect of genetic or pharmacological inhibition of iNOS on body weight**. Body weight was recorded immediately before bleomycin administration and daily for all the experimental period. The genetic or pharmacological inhibition of iNOS significantly prevents the loss of body weight. Data are means ± s.e. means from 10 mice for each group. *p < 0.01 represents significant reduction of the various parameters in the group in which iNOS was inhibited or absent.

**Figure 8:**
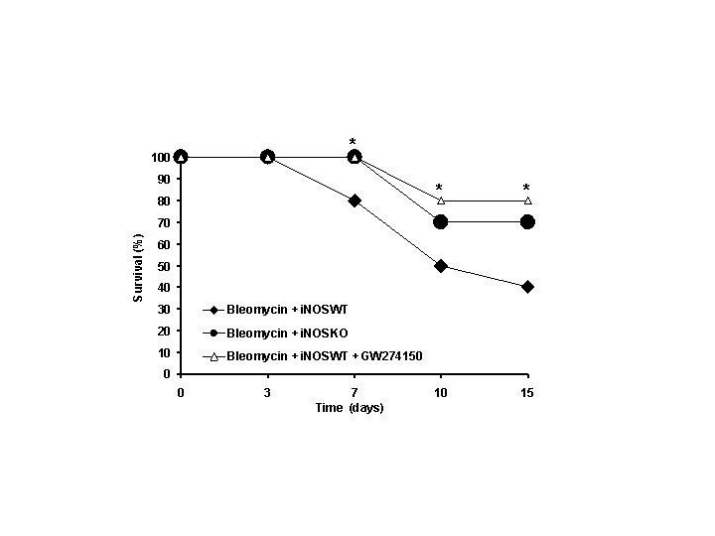
**Effect of genetic or pharmacological inhibition of iNOS on bleomycin-induced mortality**. Survival is significantly improved in iNOSKO and iNOSWT GW274150-treated mice in comparison to the high mortality rate of the bleomycin-treated iNOSWT mice. Data are means ± s.e. means from 20 mice for each group. *p < 0.01 represents significant reduction of the various parameters in the group in which iNOS was inhibited or absent.

In iNOSWT mice, the severe lung injury caused by bleomycin administration was associated with a significant loss in body weight (Fig. 7). The absence or inhibition of iNOS in mice (animals with the iNOSKO phenotype or iNOSWT mice treated with GW274150) significantly attenuate the loss in body weight (Fig. 7). The survival of animals was monitored for 15 days. Bleomycin-treated iNOSWT mice developed severe lung injury and 60% of these animals died within 15 days after bleomycin administration (Fig 8). In contrast, none of the iNOSKO mice as well as the iNOSWT which had been treated with GW274150 died (Fig. 8).

### Bronchoalveolar Lavage

**Figure 9:**
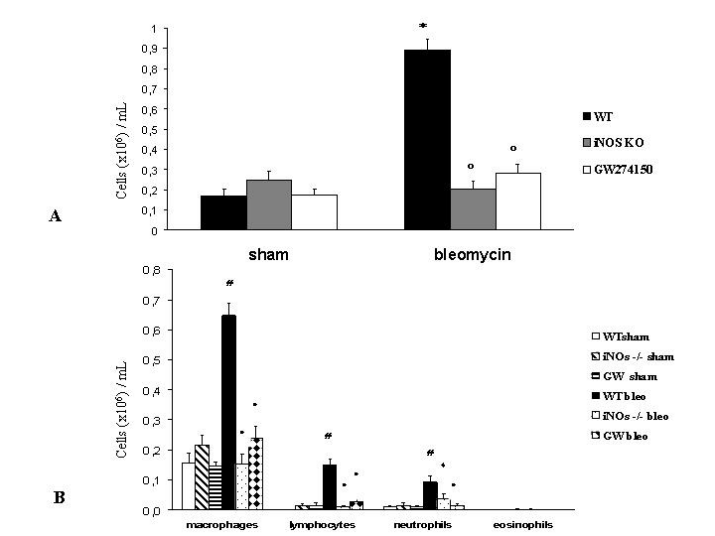
**Effect of genetic or pharmacological inhibition of iNOS on bleomycin-induced total and differential cellularity of bronchoalveolar lavage (BAL)**. **(A) **Total BAL cellularity for sham and bleomycin treated mice. **(B) **Differential cells counts for macrophages, lymphocytes, neutrophils and eosinophils per milliliter of BAL fluid are shown. Data, expressed as means ± s.e., are representative of 10 mice for each group. * p < 0.001 vs. sham, °p < 0.001 vs. bleomycin treated iNOSWT, # p < 0.001 vs. sham, ● p < 0.001 vs. bleomycin treated iNOSWT, ◆ p < 0.05 vs. bleomycin treated iNOSWT.

Instillation of saline solution produced no significant increase in leukocyte numbers in BAL fluid of iNOSKO and GW274150 treated iNOSWT mice compared to the sham wild type group (2.48 ± 0.45 and 1.77 ± 0.24 vs. 1.69 ± 0.37 cells × 10^5^/mL ± SE). Bleomycin instillation in iNOSWT mice produced a significant increase of inflammatory cells compared to sham iNOSWT mice (8.93 ± 0.53 vs 1.69 ± 0.37 cells × 10^5^/mL ± SE, p < 0.001) (Fig. 9). iNOSKO and GW274150 treated iNOSWT mice that underwent to bleomycin tracheal instillation did not show such an increase of BAL total cellularity as compared to bleomycin iNOSWT mice group (2.05 ± 0.35 and 2.83 ± 0.41 vs. 8.92 ± 0.53 cells × 10^5^/mL ± SE, p < 0.001). Differential cell counts showed a similar profile across all of the sham groups. In bleomycin treated iNOSWT mice it was evident an increase of monocytes (6.48 ± 0.39 vs. 1.57 ± 0.36 cells × 10^5^/mL ± SE, p < 0.001), lymphocytes (1.49 ± 0.20 vs. 0.19 ± 0.08 cells × 10^5^/mL ± SE, p < 0.001) and neutrophils (0.94 ± 0.20 vs. 0.10 ± 0.04 cells × 10^5^/mL ± SE, p < 0.001) if compared to sham wild type mice. iNOSKO and GW274150 treated iNOSWT mice that underwent to bleomycin tracheal instillation did not show any increase of BAL inflammatory cells (Fig. 9). In these mice monocytes (1.55 ± 0.31 and 2.40 ± 0.41 vs. 6.48 ± 0.39 cells × 10^5^/mL ± SE, p < 0.001), lymphocytes (0.11 ± 0.03 and 0.27 ± 0.1 vs. 1.49 ± 0.2 cells × 10^5^/mL ± SE, p < 0.001) and neutrophils (0.38 ± 0.18 and 0.16 ± 0.07 vs. 0.94 ± 0.20 cells × 10^5^/mL ± SE, p < 0.05 and p < 0.001 respectively) were significantly reduced compared to bleomycin treated iNOSWT group (Fig. 9). Eosinophils did not show any statistically significant difference among all groups.

### TGF-β1 western blot analysis

**Figure 10:**
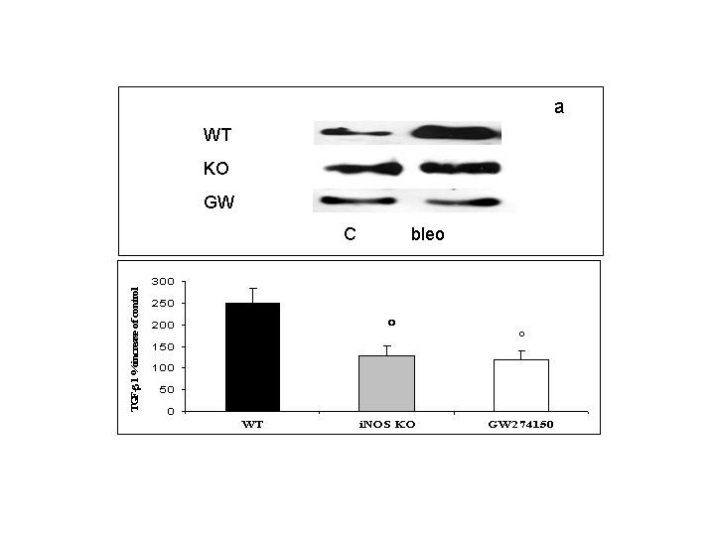
**Western blot analysis of TGF-β1 expression in wild type (WT), iNOS^-/-^(KO) and GW274150-treated (GW) mice exposed to vehicle (C) or bleomycin (bleo) for 7 days**. Representative blots are reported in a. In b, data, normalized for β-actin expression, represent mean the bleomycin induced TGF-β1 percent increase of control groups (+/- SE) of 7 independent experiments. *p < 0.05 and **p < 0.001 vs iNOSWT mice.

TGF-β1 was expressed in all sham groups undergoing intra-tracheal saline instillation as detected by western blot analysis (data not shown). Exposure of iNOSWT mice to bleomycin produced a remarkable increase of TGF-β1 expression (247.9 + 34% of control). In contrast, iNOSKO and GW274150-treated iNOSWT mice subjected to intra-tracheal bleomycin instillation exhibited only a slight increase of TGF-β1 expression, 129.4 ± + 21.4% and 120.1 ± 19.4% for iNOSKO and GW274150-treated iNOSWT mice, respectively, that yielded statistical significance when compared to iNOSWT (p < 0.05 and p < 0.001) (Fig. 10).

## Discussion

Pulmonary fibrosis is a common response to various insults to the lung and it is the end-point of a numerous and heterogeneous group of disorders known as interstitial lung diseases (ILD) that are characterized by chronic inflammation and progressive fibrosis of the pulmonary interstitium: alveolar walls (including epithelial cells and capillaries), septae, and the perivascular, perilymphatic, and peribronchiolar connective tissues [32]. While the pathogenesis is incompletely understood, a growing body of evidence suggests two different pathogenic routes for developing pulmonary fibrosis. The inflammatory pathway, where a shift to the so-called T-helper 2 type cytokine networks is critical, and the epithelial pathway represented by idiopathic pulmonary fibrosis, by far the most aggressive ILD. Both routes may trigger a number of cytokines/growth factors inducing fibroblast migration/proliferation and phenotype change to myofibroblasts, with a consequent accumulation of extracellular matrix [33]. In addition, various evidences have point out an important role for IL-6 and IL-11 in the pulmonary fibrosis [34,35].

The common pathologic features in ILD, include the fibrosis of the interstitium, involve collagen, elastic and smooth muscle elements, architectural remodeling and chronic inflammation of the interstitium (*ie*, variable increases in lymphocytes, neutrophils, plasma cells, macrophages, eosinophils, and mast cells), hyperplasia of type II cells and hyperplasia of endothelial cells [32]. Intratracheal instillation of the antitumour agent BLM is the most commonly used animal model for pulmonary fibrosis [36]. The bleomycin-oxygen complex is thought to bind to DNA and lead to the efficient cleavage of the phosphodiester-deoxyribose backbone and the generation of ROS [37] In the presence of oxygen and a reducing agent in fact, the ferrous ion-BLM complex becomes activated and functions mechanically as a ferrous oxidase, transferring electrons from ferrous ion to molecular oxygen to produce ROS that cause scission of DNA [38,39]. Therefore, earlier reports [40,41] point out that the pathogenesis of BLM-induced fibrosis, at least in part, is mediated through the generation of reactive oxygen species (ROS) which cause the peroxidation of membrane lipids and DNA damage. If, that perspective is true, then antioxidant therapy may prevent the lung fibrosis caused by BLM and may prevent other diseases related with interstitial pulmonary fibrosis. Because BLM administration results in increased lipid peroxidation (LPO) and alters activities of antioxidant enzymes in bronchoalveolar lavage fluids (BALFs) and lung tissue [42,43], in previous studies [44,45] some natural or synthetic antioxidants have been used to protect against BLM oxidative lung toxicity both in vivo and also in vitro. In addition to ROS, an overproduction of nitric oxide (NO) due to the expression of the inducible isoform of NO synthase (iNOS) also plays important role in various models of inflammation [5,9,46]. Pharmacological inhibitors of NOS, and also ablation of the gene for iNOS has been shown to reduce the development of the inflammatory response [47-49]. In addition, various studies have point out that iNOS play an important role in the pulmomary fibrosis induced by bleomycin [50-52]. Therefore, some study have also demonstrated that non-selective and partial selective iNOS inhibitor like aminoguanidine exert beneficial effect against lung injury induced by bleomycin [53-57]. However, frequently a misunderstanding is the definition of an inhibitor as selective for, e.g. iNOS versus eNOS, and then ignoring its non-selectivity for nNOS or completely distinct enzyme targets. An interesting example is aminoguanidine that is a partially selective for iNOS versus eNOS [58], while the selectivity over nNOS is minimal. Moreover it has a wide range of other effects, inhibiting advanced glycosylation end-product formation, diamine oxidase and polyamine metabolism [59,60], catalase [61] and having anti-oxidant effects [62,63]. For this reason aminoguanidine should not be described as a selective inhibitor.

In contrast, GW274150 is a novel, potent and selective inhibitor of iNOS activity and previous studies have demonstrated its protective effect in organ injury in hemorrhagic shock, in renal ischemia and reperfusion and in a model of collagen-induced arthritis [24-26]. This study provides the first evidence of the protective role of GW274150 in an experimental model of lung fibrosis. Here we demonstrate that the lack of iNOS gene as well as the pharmacological inhibition of iNOS (GW274150 treatment) reduces: i) the development of bleomycin-induced lung injury, (ii) the infiltration of the lung with inflammatory cells, (iii) the degree of nitrosative stress in the lung and (iv) the mortality rate. All of these findings support the view that NO plays an important role in the degree of inflammation and lung fibrosis caused by bleomycin in the mice. We report in the present study a reduction of the tissue damage in the lung of bleomycin-treated iNOSKO mice as well as bleomycin-treated iNOSWT mice which received the treatment with GW274150.

Two of the most recognized fibrosis markers, lung collagen deposition and TGF-β1 expression, were significantly reduced in these animals. Evidence from animal models and human studies suggests that TGF-β1 plays a central role in a variety of fibroproliferative disorders, including pulmonary fibrosis. TGF-β1 plays a critical role in the pathogenesis of lung fibrosis through stimulation of collagen and fibronectin production in fibroblasts [64], as well as through inhibition of biosynthesis of proteases that degrade the extracellular matrix [65]. TGF-β1 promotes wound healing [66] and its presence has been shown to be increased in bleomycin -induced lung fibrosis [67,68] and particularly in lung macrophages [69]. In addition it has been shown that during the course of pulmonary fibrosis the increase of TGF-β1 mRNA precede the increase of type I and type III procollagen mRNAs The secretion of biologically active TGF-β1 by alveolar macrophages is transiently elevated in bleomycin -induced pulmonary inflammation, whereas latent (L)-TGF-β1 secretion remains elevated for a prolonged length of time and it is probable that the extent of inflammation and fibrosis in this model depends on the quantity of active TGF-β1 available [70].

In BAL of iNOSWT animals, that underwent bleomycin instillation we observed, a strong increase of inflammatory cells such as macrophages and neutrophils. This could justify, at least in part, the significantly higher TGF-β1 expression and the increased lung collagen content in these mice. Bleomycin-treated iNOSKO mice as well as bleomycin-treated iNOSWT mice which received the treatment with GW274150 showed both significantly reduced TGF-β1 expression as well as lung collagen deposition, together with a significantly reduced inflammatory cells presence in the BAL. Furthermore we report in the present study in the lung tissue of bleomycin-treated iNOSKO mice as well as bleomycin-treated iNOSWT mice which received the treatment with GW274150 a significant reduction of leukocyte infiltration as assessed by the specific granulocyte enzyme MPO. Neutrophils recruited into the tissue can contribute to tissue destruction by the production of reactive oxygen metabolites, granule enzymes, and cytokines that further amplify the inflammatory response by their effects on macrophages and lymphocytes [71]. Furthermore, we found that the tissue damage induced by bleomycin in vehicle-treated mice was associated with an intense immunostaining of nitrotyrosine formation also suggesting that a structural alteration of the lung had occurred, most probably due to the formation of highly reactive nitrogen-derivatives. Recent evidence indicates, that bleomycin is a well-known cause of intracellular oxidative stress, several findings in this study suggest that extracellular oxidative stress may also play a role in the pathogenesis of bleomycin-induced lung injury [72,73]. Therefore, in this study we clearly demonstrate that the genetic and pharmacological inhibition of iNOS prevent the formation of peroxynitrite. Nitrotyrosine formation, along with its detection by immunostaining, was initially proposed as a relatively specific marker for the detection of the endogenous formation "footprint" of peroxynitrite [74]. There is, however, recent evidence that certain other reactions can also induce tyrosine nitration; e.g., the reaction of nitrite with hypoclorous acid and the reaction of myeloperoxidase with hydrogen peroxide can lead to the formation of nitrotyrosine [75]. Increased nitrotyrosine staining is considered, therefore, as an indication of "increased nitrative stress" rather than a specific marker of the generation of peroxynitrite. From the present data, we cannot determine the mechanism of tyrosine nitration: inhibition of NOS by NOS inhibitor would inhibit both NO formation (and thus, reduce the generation of peroxynitrite) as well as it would suppress nitrite formation (and thereby attenuate the peroxidase dependent mechanisms of tyrosine nitration). Nevertheless, we can certainly conclude from the current data that the absence of iNOS significantly reduced tyrosine nitration *in vivo*.

## Conclusion

In conclusion, this study demonstrates that the degree of inflammation and fibrosis caused by injection of bleomycin is significantly attenuated in iNOSKO mice as well as in the iNOSWT mice treated with GW274150. These findings support the view that the induction of iNOS contributes to the extension of inflammation in the model of bleomycin-induced lung fibrosis used here. Finally, we provide the first evidence that GW274150 causes a substantial reduction of lung fibrosis in the mice and our findings suggest that interventions, which may reduce the generation or the effects of iNOS, may be useful in conditions associated with local or systemic inflammation.

## References

[B1] Gross TJ, Hunninghake GW (2001). Idiopathic pulmonary fibrosis. N Engl J Med.

[B2] Crouch E (1990). Pathobiology of pulmonary fibrosis. Am J Physiol.

[B3] Crystal RG, Bitterman PB, Rennard SI, Hance AJ, Keogh BA (1984). Interstitial lung diseases of unknown cause: disorders characterized by chronic inflammation of the lower respiratory tract. N Engl J Med.

[B4] Sleijfer S (2001). Bleomycin-induced pneumonitis. Chest.

[B5] Nathan C (1992). Nitric oxide as a secretary product of mammalian cells. FASEB J.

[B6] Dinerman J (1993). Molecular mechanism of nitric oxide production. Potential relevance to cardiovascolar disease. Circ Res.

[B7] Szabò C (1995). Alterations in the production of NO in various forms of circulatory shock. New Horiz.

[B8] Southan G, Szabò C (1996). Selective pharmacological inhibition of distinct NOS isoforms. Bioch Pharm.

[B9] Moncada S, Palmer RMJ (1991). NO: physiology, patophysiology and pharmacology. Pharm Rev.

[B10] Moncada S, Higgs A (2002). The L-arginine NO pathway. New Eng J of Med.

[B11] Cuzzocrea S (2004). Effect of inhibitors of nitric oxide in animal models and future directions for therapy in inflammatory disorders. Current Med Chem Anti-inflammatory & Anti-allergy agent.

[B12] Crow JP, Beckman JS (1995). Reactions between nitric oxide, superoxide, and peroxynitrite: footprints of peroxynitrite in vivo. Adv Pharmacol.

[B13] Pryor WA, Squadrito GL (1995). The chemistry of peroxinitrite: a product from reaction of NO with superoxide. Am J Physiol.

[B14] Beckman JS, Beckman TW, Chen J, Marshall PA, Freeman BA (1990). Apparent hydroxil radical production by peroxynitrite: implications for endothelial injury from NO and superoxide. Proc Natl Acad Sci USA.

[B15] Cuzzocrea S, Riley DP, Caputi AP, Salvemini D (2001). Antioxidant therapy: a new pharmacological approach in shock, inflammation, and ischemia/reperfusion injury. Pharmacol Rev.

[B16] Rao AV, Balachandran B (2002). Role of oxidative stress and antioxidants in neurodegenerative diseases. Nutr Neurosci.

[B17] Iuliano L (2001). The oxidant stress hypothesis of atherogenesis. Lipids.

[B18] Yamada T, Grisham MB (1991). Role of neutrophil-derived oxidants in the pathogenesis of intestinal inflammation. Klin Wochenschr.

[B19] Inoue S, Kawanishi S (1995). Oxidative DNA damage induced by simultaneous generation of nitric oxide and superoxide. FEBS Lett.

[B20] Salgo MG (1995). Peroxinitrite causes apoptosis in rat thymorytes. Bioch Bioph Res Commun.

[B21] Szabò C (1998). Role of poly(ADP-ribose)synthetase in inflammation. Eur J Pharmacol.

[B22] Alderton WK, Cooper CE, Knowles RG (2001). Nitric oxide synthases: structure, function and inhibition. Biochem J.

[B23] Young RJ, Beams RM, Carter K, Clark HA, Coe DM, Chambers CL, Davies PI, Dawson J, Drysdale MJ, Franzman KW, French C, Hodgson ST, Hodson HF, Kleanthous S, Rider P, Sanders D, Sawyer DA, Scott KJ, Shearer BG, Stocker R, Smith S, Tackley MC, Knowles RG (2000). Inhibition of inducible nitric oxide synthase by acetamidine derivatives of hetero-substituted lysine and homolysine. Bioorg Med Chem Lett.

[B24] Mcdonald MC, Izumi M, Cuzzocrea S, Thiemermann C (2002). A novel, potent and selective inhibitor of the activity of inducible nitric oxide synthase (GW274150) reduces the organ injury in hemorrhagic shock. J Physiol Pharmacol.

[B25] Chatterjee PK, Patel NS, Sivarajah A, Kvale EO, Dugo L, Cuzzocrea S, Brown PA, Stewart KN, Mota-Filipe H, Britti D (2003). GW274150, a potent and highly selective inhibitor of iNOS, reduces experimental renal ischemia/reperfusion injury. Kidney Int.

[B26] Cuzzocrea S, Chatterjee PK, Mazzon E, Mcdonald MC, Dugo L, Di Paola R, Serraino I, Britti D, Caputi AP, Thiemermann C (2002). Beneficial effects of GW274150, a novel, potent and selective inhibitor of iNOS activity, in a rodent model of collagen-induced arthritis. Eur J Pharmacol.

[B27] Dugo L, Marzocco S, Mazzon E, Di Paola R, Genovese T, Caputi AP, Cuzzocrea S (2004). Effects of GW274150, a novel and selective inhibitor of iNOS activity, in acute lung inflammation. Br J Pharmacol.

[B28] Ashcroft T, Simpson JM, Timbrell V (1988). Simple method of estimating severity of pulmonary fibrosis on a numerical scale. J Clin Pathol.

[B29] Cuzzocrea S, Ianaro A, Wayman NS, Mazzon E, Pisano B, Dugo L, Serraino I, Di Paola R, Chatterjee PK, Di Rosa M (2003). The cyclopentenone prostaglandin 15-deoxy-delta-(12,14)-PGJ2 attenuates the development of colon injury caused by dinitrobenzene sulphonic acid in the rat. Br J Pharmacol.

[B30] Mullane KM, Kraemer R, Smith B (1985). Myeloperoxidase activity as a quantitative assessment of neutrophil infiltration into ischemic myocardium. J Pharmacol Methods.

[B31] Bradford M (1976). A rapid and sensitive method for the quantitation of microgram quantities of protein utilizing the principle of proteindye binding. Anal Biochem.

[B32] Green FH (2002). Overview of pulmonary fibrosis. Chest.

[B33] Pardo A, Selman M (2002). Molecular mechanisms of pulmonary fibrosis. Front Biosci.

[B34] Hardie WD, Le Cras TD, Jiang K, Tichelaar JW, Azhar M, Korfhagen TR (2004). Conditional expression of transforming growth factor-alpha in adult mouse lung causes pulmonary fibrosis. Am J Physiol Lung Cell Mol Physiol.

[B35] Tang W, Geba GP, Zheng T, Ray P, Homer RJ, Kuhn C, Flavell RA, Elias JA (1996). Targeted expression of IL-11 in the murine airway causes lymphocytic inflammation, bronchial remodeling, and airways obstruction. J Clin Invest.

[B36] Chandler DB, Hyde DM, Giri SN (1983). Morphometric estimates of infiltrative cellular changes during the development of bleomycin-induced pulmonary fibrosis in hamsters. Am J Pathol.

[B37] Hecht SM (2000). Bleomycin: new perspectives on the mechanism of action. J Nat Prod.

[B38] Burger RM, Projan SJ, Horwitz SB, Peisach J (1986). The DNA cleavage mechanism of iron-bleomycin. Kinetic resolution of strand scission from base propenal release. J Biol Chem.

[B39] Arslan SO, Zerin M, Vural H, Coskun A (2002). The effect of melatonin on bleomycin-induced pulmonary fibrosis in rats. J Pineal Res.

[B40] Slosman DO, Costabella PM, Roth M, Werlen G, Polla BS (1990). Bleomycin primes monocytes-macrophages for superoxide production. Eur Respir J.

[B41] Goodman MT, Hernandez B, Wilkens LR, Lee J, Le Marchand L, Liu LQ, Franke AA, Kucuk O, Hsu TC (1998). Effects of beta-carotene and alpha-tocopherol on bleomycin-induced chromosomal damage. Cancer Epidemiol Biomarkers Prev.

[B42] Giri SN, Chen ZL, Younker WR, Schiedt MJ (1983). Effects of intratracheal administration of bleomycin on GSH-shuttle enzymes, catalase, lipid peroxidation, and collagen content in the lungs of hamsters. Toxicol Appl Pharmacol.

[B43] Karam H, Hurbain-Kosmath I, HousSET B (1998). Antioxidant activity in alveolar epithelial type 2 cells of rats during the development of bleomycin injury. Cell Biol Toxicol.

[B44] Ikezaki S, Nishikawa A, Enami T, Furukawa F, Imazawa T, Uneyama C, Fukushima S, TakahASHI M (1996). Inhibitory effects of the dietary antioxidants butylated hydroxyanisole and butylated hydroxytoluene on bronchioloalveolar cell proliferation during the bleomycin-induced pulmonary fibrosing process in hamsters. Food Chem Toxicol.

[B45] Venkatesan N, Punithavathi V, Chandrakasan G (1997). Curcumin protects bleomycin-induced lung injury in rats. Life Sci.

[B46] Cuzzocrea S, Zingarelli B, Gilard E, Hake P, Salzman AL, Szabó C (1998). Anti-inflammatory effects of mercaptoethylguanidine, a combined inhibitor of nitric oxide synthase and peroxynitrite scavenger, in carrageenan-induced models of inflammation. Free Rad Biol Med.

[B47] Salvemini D, Wang ZQ, Wyatt P, Bourdon DM, Marino MH, Manning PT, Currie MG (1996). Nitric oxide: a key mediator in the early and late phase of carrageenan-induced rat paw inflammation. Br J Pharmacol.

[B48] Tracey WR, Nakane M, Kuk J, Budzik G, Klinghofer V, Harris R, Carter G (1995). The nitric oxide synthase inhibitor, L-N^G^-monomethylarginine, reduces carrageenan-induced pleurisy in the rat. J Pharmacol Exp Ther.

[B49] Wei XQ, Charles IG, Smith A, Ure J, Feng GJ, Huang FP, Xu D, Muller W, Moncada S, Liew FY (1995). Altered immune responses in mice lacking inducible nitric oxide synthase. Nature.

[B50] El-Khatib AS (2002). Possible modulatory role of nitric oxide in lung toxicity induced in rats by chronic administration of bleomycin. Chemotherap.

[B51] Jang AS, Lee JU, Choi IS, Park KO, Lee JH, Park SW, Park CS (2004). Expression of nitric oxide synthase, aquaporin 1 and aquaporin 5 in rat after bleomycin inhalation. Intensive Care Med.

[B52] Davis DW, Weidner DA, Holian A, McConkey DJ (2000). Nitric oxide-dependent activation of p53 suppresses bleomycin-induced apoptosis in the lung. J Exp Med.

[B53] de Rezende MC, Martinez JA, Capelozzi VL, Simoes MJ, Beppu OS (2000). Protective effect of aminoguanidine in a murine model of pulmonary fibrosis induced by bleomycin. Fundam Clin Pharmacol.

[B54] Hu J, Xu Q, Li B (1999). The effect of aminoguanidine, a nitric oxide synthase inhibitor, on bleomycin-induced lung injury in rats. Zhonghua Jie He He Hu Xi Za Zhi.

[B55] Yildirim Z, Turkoz Y, Kotuk M, Armutcu F, Gurel A, Iraz M, Ozen S, Aydogdu I, Akyol O (2004). Effects of aminoguanidine and antioxidant erdosteine on bleomycin-induced lung fibrosis in rats. Nitric Oxide.

[B56] Giri SN, Biring I, Nguyen T, Wang Q, Hyde DM (2002). Abrogation of bleomycin-induced lung fibrosis by nitric oxide synthase inhibitor, aminoguanidine in mice. Nitric Oxide.

[B57] Chen XL, Huang SS, Li WB, Wang DH, Wang XL (2001). Inhibitory effect of aminoguanidine on bleomycin-induced pulmonary toxicity in rat. Acta Pharmacol Sin.

[B58] Alderton WK, Cooper CE, Knowles RG (2001). NO Synthases: Structure, Function and Inhibition. Biochem J.

[B59] Bieganski T, Kusche J, Lorenz W, Hesterberg R, Stahlknecht CD, Feussner KD (1983). Distribution and Properties of Human Intestinal Diamine Oxidase and Its Relevance for the Histamine Catabolism. Biochim Biophys Acta.

[B60] Nilsson BO, Kockum I, Rosengren E (1996). Inhibition of Diamine Oxidase Promotes Uptake of Putrescine From Rat Small Intestine. Inflamm Res.

[B61] Ou P, Wolff SP (1993). Aminoguanidine: a Drug Proposed for Prophylaxis in Diabetes Inhibits Catalase and Generates Hydrogen Peroxide in Vitro. Biochem Pharmacol.

[B62] Giardino I, Fard AK, Hatchell DL, Brownlee M (1998). Aminoguanidine Inhibits Reactive Oxygen Species Formation, Lipid Peroxidation, and Oxidant-Induced Apoptosis. Diabetes.

[B63] Yildiz G, Demiryurek AT, Sahin-Erdemli I, Kanzik I (1998). Comparison of Antioxidant Activities of Aminoguanidine, Methylguanidine and Guanidine by Luminol-Enhanced Chemiluminescence. Br J Pharmacol.

[B64] Fine A, Goldstein RH (1987). The effect of transforming growth factor on cell proliferation and collagen by fibroblasts. J Biol Chem.

[B65] Sporn MB, Roberts AB, Wakefield LM, De Crombrugghe B (1987). Some recent advances in the chemistry and biology of transforming growth factor-beta. J Cell Biol.

[B66] Pierce GF, Mustoe TA, Lingelbach J, Masakowski VR, Gramates P, Deuel TF (1989). Transforming growth factor reverses the glucocorticoid-induced wound-healing deficit in rats: possible regulation in macrophages by platelet-derived growth factor. Proc Natl Acad Sci USA.

[B67] Hoyt DG, Lazo JS (1988). Alterations in pulmonary mRNA encoding procollagens, fibronectin and transforming growth factor precede bleomycin induced pulmonary fibrosis in mice. J Pharmacol Exp Ther.

[B68] Raghow R, Irish P, Kang AH (1989). Coordinate regulation of transforming growth factor gene expression and cell proliferation in hamster lungs undergoing bleomycin-induced pulmonary fibrosis. J Clin Invest.

[B69] Khalil N, Bereznay O, Sporn M, Greenberg AH (1989). Macrophage production of transforming growth factor and fibroblast collagen synthesis in chronic pulmonary inflammation. J Exp Med.

[B70] Khalil N, Corne S, Whitman C, Yacyshyn H (1996). Plasmin regulates the activation of cell-associated latent TGF-1 secreted by rat alveolar macrophages after in vivo bleomycin injury. Am J Respir Cell Mol Biol.

[B71] Chatham WW, Swaim R, Frohsin H, Heck LW, Miller EJ, Blackburn WD (1993). Degradation of human articular cartilage by neutrophils in synovial fluid. Arthritis Rheum.

[B72] Bowler RP, Nicks M, Warnick K, Crapo JD (2002). Role of extracellular superoxide dismutase in bleomycin-induced pulmonary fibrosis. Am J Physiol Lung Cell Mol Physiol.

[B73] Chen XL, Li WB, Zhou AM, Ai J, Huang SS (2003). Role of endogenous peroxynitrite in pulmonary injury and fibrosis induced by bleomycin A5 in rats. Acta Pharmacol Sin.

[B74] Beckman JS (1996). Oxidative damage and tyrosine nitration from peroxynitrite. Chem Res Toxicol.

[B75] Eiserich JP, Hristova M, Cross CE, Jones AD, Freeman BA, Halliwell B, Van der Vliet A (1998). Formation of nitric oxide-derived inflammatory oxidants by myeloperoxidase in neutrophils. Nature.

